# Supplementary material for: Content-rich biological network constructed by mining PubMed abstracts
Source: BMC Bioinformatics. 2004 Oct 8;5:147. doi: 10.1186/1471-2105-5-147 (PMC528731; doi:10.1186/1471-2105-5-147)
Supplement: Additional File 2 — The original results of the above study (non-essential files are deleted to keep the file size under the limit set by BMC bioinformatics). [file 1471-2105-5-147-S2.bz2 › chilibotAdditionalFile2/dip05/647ID9846874E11034/html/CDC42_ARHGAP1.html]

 


 **CDC42** and **ARHGAP1** 
  
Found 46 abstracts in PubMed, retrieved 05.  
 

 What does Google say? 
 PDF only 
| .edu only 

---

**Interactive relationship** (e.g. stimulation, inhibition, etc)

**Neutral relationship**- Together, these findings indicate that during cytokinesis, MgcRacGAP, previously known as a GAP for Rac  **Cdc42** , is functionally converted to a RhoGAP  [ **ARHGAP1** ]  through phosphorylation by Aurora B.  Ref: 12689593 Dev Cell, 2003

**Non-interactive relationship** (e.g. studied together, co-existance, homology, etc.)

- While ExoT RhoGAP  [ **ARHGAP1** ]  stimulates actin reorganization through the inactivation of Rho, Rac and  **Cdc42** , the function of the ADP ribosylation domain is unknown.  Ref: 12807879 J Biol Chem< MedlineTA>J Biol Chem, 2003
